# L-Glutamine Supplementation Prevents the Development of Experimental Diabetic Cardiomyopathy in Streptozotocin-Nicotinamide Induced Diabetic Rats

**DOI:** 10.1371/journal.pone.0092697

**Published:** 2014-03-20

**Authors:** Sachin L. Badole, Ganesh B. Jangam, Swapnil M. Chaudhari, Arvindkumar E. Ghule, Anand A. Zanwar

**Affiliations:** 1 Department of Pharmacology, PES's Modern College of Pharmacy, Yamuna Nagar, Nigadi, Pune, India; 2 Department of Pharmacology, Poona College of Pharmacy, Bharati Vidyapeeth Deemed University, Erandwane, Pune, India; 3 Center for Innovation in Nutrition Health Disease, Interactive Research School for Health Affairs, Medical college campus, Bharati Vidyapeeth Deemed University, Dhankawadi, Pune, India; University of Florida, United States of America

## Abstract

The objective of the present investigation was to evaluate the effect of L-glutamine on cardiac myopathy in streptozotocin-nicotinamide induced diabetic rats. Diabetes was induced in overnight fasted Sprague Dawely rats by using intraperitonial injection of streptozotocin (55 mg/kg). Nicotinamide (100 mg/kg, i.p.) was administered 20 min before administration of streptozotocin. Experimental rats were divided into Group I: non-diabetic control (distilled water; 10 ml/kg, p.o.), II: diabetic control (distilled water, 10 ml/kg, p.o.), III: L-glutamine (500 mg/kg, p.o.) and IV: L-glutamine (1000 mg/kg, p.o.). All groups were diabetic except group I. The plasma glucose level, body weight, electrocardiographic abnormalities, hemodynamic changes and left ventricular contractile function, biological markers of cardiotoxicity, antioxidant markers were determined after 4 months after STZ with nicotinamide injection. Histopathological changes of heart tissue were carried out by using H and E stain. L-glutamine treatment improved the electrocardiographic, hemodynamic changes; LV contractile function; biological markers; oxidative stress parameters and histological changes in STZ induced diabetic rats. Results from the present investigation demonstrated that L-glutamine has seemed a cardioprotective activity.

## Introduction

Diabetic cardiomyopathy is defined as the changes induced by diabetes mellitus in cardiac structure, ventricular dysfunction of ischemic heart disease, hypertension or cardiac pathologies. Diabetic patients are at an increased risk of cardiovascular diseases and these are the major cause of death in them. Cardiac dysfunction occurs at six to twelve weeks even early at 2 weeks onset of diabetes [1], [2].

L-glutamine (Gln) is among the 20 amino acids encoded by the standard genetic code. It is the most abundant free amino acid of the human body [3]. It comprises about 20% of free amino acids in plasma and more than 50% of the amino acid pool in human skeletal muscle [4]. It plays role in the maintenance and function of many organs and tissues such as the kidneys, liver, intestine, heart, muscle, neurons, lymphocytes, macrophages, neutrophils and pancreatic β-cells [5]. L-glutamine improves cardiac function in patients with acute myocardial infarction [6].

Recently, Menge *et al.*, (2010) reported that the selective deficiencies of various amino acids including glutamine and arginine in patients with type 2 diabetes may argue in favour of a selective amino acid supplementation in diabetic patients [7]. Also administration of glutamine (i.p.) resulted in increased heat shock protein 70 (HSP 70) expression as a cardioprotective mechanism in left heart tissues in the presence of diabetes mellitus in rats [8]. Also, Tsai et al., (2011) reported that supplemental dietary glutamine increased the antioxidant potential and consequently decreased leukocyte adhesion molecule expression and oxidative stress in organs of mice with type 1 diabetes [9]. Oral administration of L-glutamine in doxorubicin and isoprenaline-induced cardiotoxicity is also known [10], [11]. Previously reported data indicated that L-glutamine produced crucial role in diabetes and cardiovascular disorders. However there is paucity of reports on effect of L-glutamine in diabetic complications. Hence objective of the present study was to investigate the effect of L-glutamine on cardiomyopathy in streptozotocin- nicotinamide induced diabetes in rats.

## Materials and Methods

### Drugs and chemicals

Streptozotocin (STZ), nicotinamide (NTM), L- glutamine, epinephrine hydrochloride, super oxide dismutase (SOD) and malondialdehyde (MDA) were purchased from Sigma chemical co. USA. Reduced glutathione (GSH), 5, 5′-dithiobis (2-nitro benzoic acid) (DTNB) (Hi media; India), thiobarbituric acid (TBA), thiopentone sodium injection (Thiosol sodium) (Neon Laboratories Ltd, India), creatine kinase-MB isoenzyme (CKMB) (Randox Laboratory, UK), LDH (Ecoline, Merck, India) and GOD/POD kit (Acuurex, India) were purchased from individual vendors. All chemicals used were of analytical grade.

### Animals and research protocol approval

Male Sprague-Dawley (SD) rats (200–250 g) were procured at our institution animal house facility, Pune, India and housed in an air-conditioned room at a temperature of 25±2 °C and relative humidity of 45% to 55% under 12-h light: 12-h dark cycle. The animals had free access to food pellets (Chakan Oil Mills, Pune, India) except when starvation was required. Water was provided *ad libitum*. The experimental protocol was approved by the Institutional Animal Ethics Committee (IAEC) constituted in accordance with the rules and guidelines of the Committee for the Purpose of Control and Supervision on Experimental Animals (CPCSEA), New Delhi, India (protocol no. MCP/IAEC/23/2011).

### Induction of diabetes

Streptozotocin was dissolved in citrate buffer (pH 4.5) and nicotinamide was dissolved in normal physiological saline. Diabetes was induced in overnight fasted Sprague Dawely rats by using intraperitonial injection of streptozotocin (55 mg/kg). Nicotinamide (100 mg/kg, i.p.) was administered 20 min before administration of streptozotocin [16]. Hyperglycaemia was confirmed after 3 days using the glucose oxidase peroxidase (GOD/POD) method. Glycemic level control of diabetic rats was made with subcutaneous injections of exogenous human NPH insulin (2 U/day of NPH insulin). All rats survived the streptozotocin and nicotinamide injection and that following hypoglycemia in all injected rats, only rats with a glucose level higher than 300 mg/dl were further used in this study.

### Effect of chronic administration of L-glutamine in diabetic rats

The rats were divided into following groups (n = 6) viz; Group I: non-diabetic control (distilled water; 10 ml/kg, p.o.), II: diabetic control (distilled water, 10 ml/kg, p.o.), III: L-glutamine (500 mg/kg, p.o.) and IV: L-glutamine (1000 mg/kg, p.o.). All groups were diabetic except group I. L-glutamine was dissolved in distilled water and administered for 4 month (once a day) at pre-determined time (15 days after injection of streptozotocin and nicotinamide). The condition of the animals was monitored daily at the time of dosing as per experimental design. Blood was collected using the retro-orbital plexus of each rat under mild ether anesthesia. Effect of anesthesia was sufficient to withdraw blood and animals were immediately recovered. Plasma glucose was thereafter determined by using GOD/POD method at end of the study period. During the study period of 4 months body weight of each rats were recorded daily but data is presented only at end of study.

### Assessment of electrocardiographic, hemodynamic changes and left ventricular (LV) contractile function

At the end of the study period, animals were anaesthetized by urethane (1.25 g/kg, i.p.). Since urethane anesthesia has minimal effects on the cardiovascular and respiratory systems and long lasting anesthesia with rapid onset following i.p. administration. The time of anesthesia is sufficient to carry out surgical procedure and record hemodyanamic and ECG parameters. Blood pressure was measured by means of a polyethylene cannula (PE 50) filled with heparinised saline (100 IU/ml) and connected to pressure transducer. The cannula was connected to a transducer and the signal was amplified by means of a bioamplifier. The right carotid artery of rat was cannulated for the measurement of systolic blood pressure (SBP), diastolic blood pressure 128 (DBP) and mean arterial blood pressure (MABP). Simultaneously for the measurement of electrocardiogram and heart rate, the leads were placed on the right foreleg (negative electrode), left foreleg (positive electrode) and right hind leg (neutral electrode). Further left ventricular systolic pressure was measured by means of a Millar mikro-tip transducer catheter (Model SRP-320, Millar instrument, Texas) inserted into the left ventricle via the right carotid artery and connected to a bioamplifier. dP/dt_max_, dP/dt_min_ and LV end diastolic pressure signals were obtained from primary signals (LV systolic pressure and blood pressure). Electrocardiographic, hemodynamic changes and left ventricular (LV) contractile function were recorded by eight channel recorder Power lab having LABCHART-6 pro software by means of an acquisition data system (AD Instruments Pty Ltd with LABCHART 7 pro software, Australia).

### Biological markers of cardiotoxicity

Serum levels of aspartate aminotransferase (AST), creatine kinase-MB isoenzyme (CKMB) and lactate dehydrogenase (LDH) enzymes were measured by automated chemistry analyzer (Micro lab 300, Merck, USA) using reagent kits.

### Effect on enzymatic biomarkers of oxidative stress

The animals were humanely euthanized after recoding all parameters by cervical dislocation method and the heart was removed and divided into two portions. One portion was used for measurement of myocardial endogenous antioxidant enzymes. Heart tissues were isolated from all rats and were cut into small pieces, placed in chilled 0.25 M sucrose solution and blotted on a filter paper. The tissues were then homogenized in 10% chilled tris hydrochloride buffer (10 mM, pH 7.4) by tissue homogenizer (Remi Motors, India) and centrifuged at 12000 r.p.m. for 15 min 0 °C using Eppendorf 5810-R high speed cooling centrifuge. The endogenous antioxidant enzymes estimated were malondialdehyde, reduced glutathione, superoxidase dismutase etc. Malondialdehyde content in supernatant of the rat heart was determined by method of Slater and Sawyer (1971) [12]. The assay of reduced glutathione was carried out by method of Moron et al. (1979) [13]. The superoxidase dismutase activity was determined by the method of Misera and Fridovich (1972) [14]. Protein concentrations were determined using the method Lowry et al. (1951) [15].

### Histopathological studies

The second portion of isolated heart were trimmed into small pieces and preserved in 10% formalin for 24 h. Specimens were cut in section of 3–5 μm in thickness and stained by hematoxyline-eosin stain. The specimen was mounted by disterene phthalate xylene (D.P.X). The photomicrographs of each tissue section were observed using Cell imaging software for Life Science microscopy (Olympus soft imaging solution GmbH, Munster, Germany-magnification: 40×). The grading system used for assessment of parameters was [–: absence of change; +: 0–30% area shows changes; + +: 30–60% area shows changes; + + +: 60–100 area shows changes].

### Statistical analysis

The data was expressed as mean ± standard error of mean (SEM). One way analysis of variance (ANOVA) was applied to test the significance of difference between average biochemical and ECG parameters of different groups and multiple comparisons were determined by *post hoc* Dunnetts test. The statistical analysis was performed using GraphPad Prism 5.0 software (Graph pad software, San Diego, California, USA). *p*<0.05 was considered statistically significant.

## Results

### Effect on plasma glucose and body weight

Repeated administration (once a day for 4 month) of L-glutamine (500 or 1000 mg/kg) caused a significant (*p*<0.001) reduction in the plasma glucose compared to diabetic control group. There was no significance difference found in vehicle (distilled water) treated animals (Table 1). Body weight of diabetic control significantly (*p*<0.001) decreased during study period compared to non-diabetic group. However, animals treated with L-glutamine (500 and 1000 mg/kg) significantly prevented this decrease (Table 1). Although L-glutamine (1000 mg/kg) was more effective in reducing plasma glucose level and body weight as compared to 500 mg/kg dose of L-glutamine. The level of significance was *p*<0.001 between L-glutamine (1000 mg/kg) and L-glutamine (500 mg/kg).

**Table 1:** Effect of L-glutamine on electrocardiographic, hemodynamic, Left ventricular function parameters and heart antioxidant enzymes in STZ-nicotinamide induced cardiomyopathy in rat.

Groups/Parameters	Control	Diabetic control	L-glutamine_(500 mg/kg)_	L-glutamine _(1000 mg/kg)_
**BSL** (mg/dl)	106.00±17.38	595.17±14.12^###^	327.43±11.75	222.17±9.92^***$$$^
**Body weight** (g)	462.32±4.32	179.85± 7.38^###^	243.27±5.21^***^	371.13±6.39^***$$$^
**Electrocardiographic**				
Heart rate (Beats/minutes)	368.48±5.45	299.95±11.16^##^	328.34±10.63^ns^	348.78±18.31^ns^
QRS Interval (ms)	20.08±1.60	14.75±0.62^##^	17.76±0.76^ns^	16.33±0.95^ns^
QT Interval (ms)	62.67±2.47	84.00±3.86^###^	72.91±1.49	69.89±2.96^*^
QTc (ms)	138.99±11.54	190.5±16.30^#^	148.35±4.78^*^	142.58±3.56^*^
SBP (mmHg)	119.58±2.46	93.31±3.95^###^	106.07±1.81^**^	115.91±0.59^***^
DBP (mmHg)	88.63±1.86	71.75±1.81^###^	78.25±2.49^ns^	85.46±2.15^***^
MABP (mmHg)	119.06±12.81	83.17±1.69^##^	89.03±1.75^ns^	104.06±2.48^ns^
**Left ventricular function**				
EDP (mmHg)	6.84±0.51	15.78±1.16^###^	11.22±0.65^**^	9.78±0.57^***^
Max dP/dt (mmHg/s)	3722.04±223.2	2472.0±91.01^###^	3129.2±110.7^ns^	3337.9±258.12
Contractility Index (1/s)	59.84±2.20	31.25±1.56^###^	44.28±2.65^**^	48.22±2.48^***^
Min dP/dt (mmHg/s)	−2477.86±162.39	−1790.0±155.71^#^	−2304±123.87^ns^	−2244.5±140.19^ns^
**Cardiac biomarkers**				
CKMB (IU/L)	1087.20±114.87	1851.94±154.54^###^	1507±98.540	1248.29±76.540^**^
LDH (IU/L)	1161±118.54	2734±97.870^###^	2184±159.08^*^	1560±123.07^***$^
AST (IU/L	154.24±19.760	482.63±54.980^###^	362.47±43.870	230.50±21.760^***^
**Oxidative stress markers**				
SOD (U/mg protein)	9.06±0.51	4.14±1.04^##^	7.82±0.64^*^	8.87±1.01^**^
GSH (μg of GSH/mg protein)	35.73±3.10	22.0±1.69^##^	33.95±1.52^*^	25.60±3.34^ns^
MDA (nM of MDA/mg protein)	2.99±0.60	4.71±0.15^##^	3.28±0.13^*^	2.94±0.092^**^

**Foot note:** Results are represented as mean ± SEM, (n = 6). Data was analyzed by one way ANOVA followed by *post hoc* Tukey's test, ^*^ *p*<0.05, ^**^ *p*<0.01, ^***^ *p*<0.001 and ns –non-significant compared with diabetic control group, ^#^ *p*<0.05, ^##^ *p*<0.01, 442 ^###^ *p*<0.001 and ns – non-significant when compared with control non-diabetic group. ^$^ *p*<0.05, ^$$^ *p*<0.001 compared with L-glutamine (500 mg/kg).

### Effect on electrocardiographic parameters

**Figure 1:**
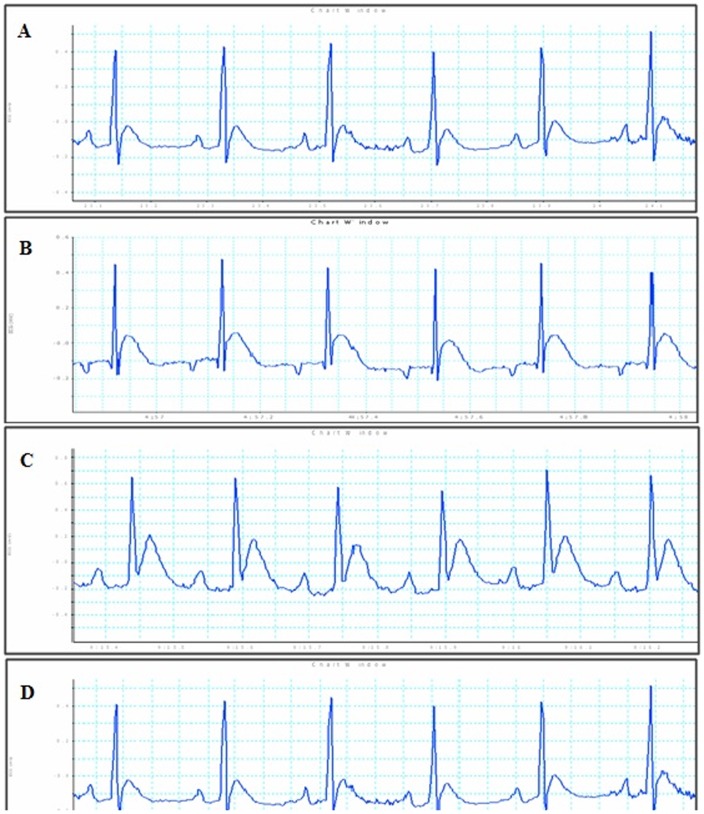
Effect on electrocardiographic parameters. (A) Non-diabetic control, (B) Diabetic control group, (C) L-glutamine (500 mg/kg) and (D) L-glutamine (1000 mg/kg).

Diabetic control rat after 4 months showed significant (*p*<0.01) reduction in heart rate compared to non-diabetic group and however treatment with L-glutamine showed non-significant change in heart rate compared to diabetic control group. Diabetic control group showed significant (*p*<0.01) decrease in QRS interval; while significant increase in QT (*p*<0.001) and QTc (*p*<0.05) interval compared to non-diabetic group. Administration of L-glutamine (1000 mg/kg) significantly (*p*<0.05) reduced QT interval as compared to diabetic group. In case of QTc interval, both L-glutamine (1000 mg/kg) and (500 mg/kg) showed significant decrease (*p*<0.05) compared to diabetic group. Administration of L-glutamine does not shown significant change in case of QRS interval compared to diabetic control group (Fig. 1 and Table 1).

### Effect on haemodynamic parameters

Diabetic group showed significant decrease in SBP (*p*<0.001), DBP (*p*<0.001), MABP (*p*<0.01), max dP/dt (*p*<0.001), contractility index (*p*<0.001) and min dP/dt (*p*<0.05) while EDP showed significant (*p*<0.001) increase in diabetic control group compared to non-diabetic group. Treatment with L-glutamine (1000 mg/kg) showed significant increase in SBP (*p*<0.001), DBP (*p*<0.01), EDP (p<0.001), dP/dtmax (*p*<0.05), contractility index (*p*<0.001), whereas non-significant change with respect to MABP and dP/dtmin were observe compared to diabetic control group (Table 1).

### Effect on cardiac biomarkers

Diabetic group significantly increase in CK-MB (*p*<0.001), LDH (*p*<0.001) and AST (*p*<0.001) compared with non-diabetic group. Treatment with L-glutamine (1000 mg/kg) significantly decreased in CK-MB (*p*<0.01), LDH (*p*<0.001) and AST (*p*<0.001) compared to diabetic control group (Table 1).

### Effect on oxidative stress parameters

In diabetic control group, the activity of superoxide dismutase (*p*<0.001) and reduced glutathione (*p*<0.01) significantly decreased and malondialdehyde (*p*<0.001) was significantly increased compare to non-diabetic groups. L-glutamine (1000 mg/kg) treatment significantly restored the contents of malondialdehyde (*p*<0.001) and superoxidase dismutase (*p*<0.01) in heart homogenate compared to diabetic group. However non-significant difference observed in reduced glutathione in L-glutamine treatment. These data indicated that, L-glutamine (1000 mg/kg) treatment improved the levels of endogenous antioxidant enzymes (SOD, GSH and MDA) in heart (Table 1).

### Histopathology rat heart

**Figure 2:**
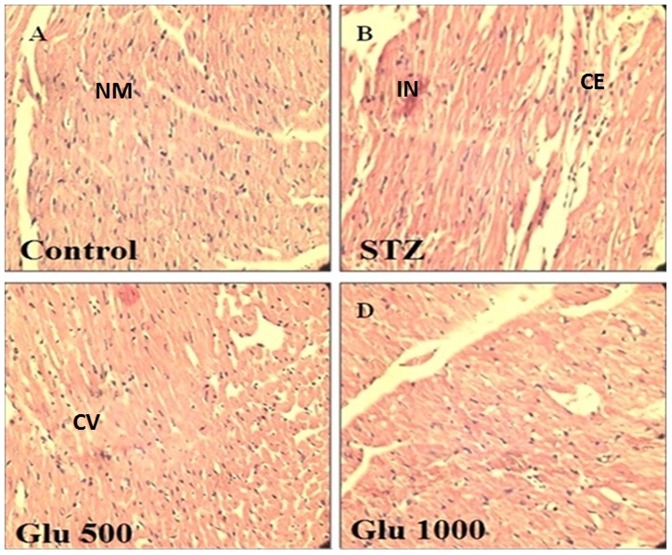
Histopathology of rat heart by hematoxyline and Eosin staining. NM =  normal myocardium, CE =  cytoplasmic eosinophilia, IN  =  mycardial inflammation, CV =  cytoplasmic vacuolization. (A) Non-diabetic control, (B) Diabetic control group, (C) L-glutamine (500 mg/kg) and (D) L-glutamine (1000 mg/kg).

Figure 2 depicts histopathological analysis by hematoxylin and eosin staining in control rats (A), diabetic control rat (B), in rat that received as L-glutamine (500 mg/kg) (C) and in rat that received as L-glutamine (1000 mg/kg) (D). The histopathology of the heart of the control group showed absence of cytoplasmic eosinophilia and myocardial inflammation (graded as −: absence of changes) indicating normal architecture of heart (Fig. 2A). Diabetic control rat showed cytoplasmic eosinophilia (++) and myocardial inflammation (++) (Fig. 2B) which means 30–60% area of heart was damaged and confirmed cardiotoxicity in rat heart (Fig. 2B). L-glutamine (500 mg/kg) showed cytoplasmic eosinophilia (++, 30–60%) and myocardial inflammation (+) mean inflammation was reduced to 0–30% and cytoplasmic eosinophilia remains unaltered (Fig. 2C). On other hand L-glutamine (1000 mg/kg) showed cytoplasmic eosinophilia (+, 0–30%) and myocardial inflammation (-), means inflammation was least/absent and cytoplasmic eosinophilia was upto 30% only (Fig. 2D). In conclusion, L-glutamine (1000 mg/kg) showed better protection with respect to these pathological changes compared to diabetic control rats indicated protective effect of L-glutamine.

## Discussion

Diabetic cardiomyopathy is characterized by an early diastolic and later systolic dysfunction with intracellular retention of calcium and sodium and loss of potassium [17]. In present study, there is alternation in haemodynamic parameters studied 4 months after induction of diabetes, suggesting cardiomyocyte damage caused by diabetic hyperglycemia. We observed that oral administration of L-glutamine for 4 month significantly reduced hyperglycaemia in streptozotocin-nicotinamide induced diabetes in SD rats. L-glutamine treatment prevented the decrease in body weight and no change in body weight was found throughout the study period (4 months). Similar resulted observed in our previous study. Administration of L-glutamine (500 or 1000 mg/kg) for 8 weeks; prevented the decrease in body weight of streptozotocin- nicotinamided induced diabetes in rats [16].

Autonomic neuropathy is common complication of both type 1 and type 2 diabetes mellitus. QT interval prolongation is important manifestation of diabetic neuropathy and has been used to screen diabetic patients at risk for sudden cardiac death [18]. The electrocardiogram showed that the QT period was extended in diabetic cardiomyopathy [17]. QTc represents corrected QT interval [18]. In present study, ECG of diabetic control showed prolonged QT and QTc interval compared with non-diabetic group whereas L-glutamine protected the cardiac function by significantly reducing the QT and QTc interval compared with diabetic control. QRS complex reflects the rapid depolarization of the right and left ventricles [18]. QRS complex was found to be decreased in diabetic control compared to non-diabetic and L-Glutamine shown non-significant change in QRS complex compared to diabetic control. Diabetic control group showed reduced systolic blood pressure, diastolic blood pressure and mean arterial blood pressure. L-glutamine treated animals showed significant improvement in systolic blood pressure and end diastolic blood pressure indicating marked cardio-protection supporting the results reported by Yan et al., (2012) where in L-glutamine shown protective effect with respect to cardiac function after myocardial damage in rats after severe burn injury [19]. As reported; *in vivo* LV cannulation was performed showed a decrease in LV dP/dtmax and dP/dtmin after 4 months of STZ injection in rats [20]. We observed that L-glutamine treated diabetic rats showed non-significant change in the rate of relaxation (dP/dtmin) and significant (*p*<0.05) in improving rate of contraction (dP/dtmax). This result suggests that L-glutamine (1000 mg/kg) provides sufficient contractile reserve to alleviate the detrimental effects of diabetes on cardiac contractility. Overall, L-glutamine significantly (*p*<0.001) improves contractility index.

It is well known that, diagnosis of cardiac enzymes is important. Aspartate aminotransferase (AST), creatine kinase-isoenezyme (CK-MB) and lactate dehydrogenase (LDH) are often used as markers of myocardial infarction [21]. Serum creatine phosphokinase activity is a more sensitive indicator in early stage of myocardial ischemia, while peak rises in lactate dehydrogenase is roughly proportional to the extent of injury to the myocardial tissue. Also, the integrity of the cardiac apparatus in drug biotransformation and metabolism could be assessed by evaluating the levels of aspartate aminotransferase, creatine kinase-MB and lactate dehydrogenase in serum [22]. In present study, L-glutamine significantly reduced the levels of cardiac enzymes such as CK-MB, LDH and AST compared with diabetic control indicating marked cardioprotection against STZ-nicotinamide induced cardiomyopathy.

Oxidative damage, inflicted by exceeding reactive oxygen species (ROS), is considered as an important pathophysiological condition, promoting cell injury and death in a broad variety [23]. Superoxide dismutase (SOD) is considered a primary enzyme since it is involved in the direct elimination of reactive oxygen species. SOD is an important defense enzyme which catalyzes the dismutation of superoxide radicals. Diabetes leads to decrease in levels of superoxide dismutase [24]. ROS degrade polyunsaturated lipids, forming malondialdehyde [25]. Diabetes leads to increase in levels of malondialdehyde. Glutathione, a tripeptide present in millimolar concentrations in all the cells, is an important antioxidant. Reduced glutathione normally plays the role of an intracellular radical scavenger and is the substrate of many xenobiotic elimination reactions. Diabetes leads to decrease in levels of reduced glutathione [16]. In present study, increase in malondialdehyde; decreased in reduced glutathione and superoxidase dismutase in heart tissue of diabetic control rats indicated an increase in oxidative stress while antioxidant activity seen in L-glutamine treated rats. Similar results observed in our previous study; oral administration of L-glutamine decreased oxidative stress in streptozotocin-nicotinamide induced diabetic rats [16]. The ratio of reduced glutathione to oxidised glutathione is the most important regulator of the redox potential. Hence L-glutamine administration may have increased the reduced glutathione to oxidised glutathione ratio, which may result in prevention of NF-kB activation and may consequently have reduced adhesion molecules expression and neutrophil infiltration to the organ [9].

Histopathological studies reported that a mild endomyocardial necrosis was present with 8 weeks of diabetes and a severe focal endomyocardial necrosis was found after 12 weeks of diabetes [26]. In present study; histological changes in diabetic heart showed cytoplasmic eosinophilia along with myocardial inflammation in after 4 month diabetic control rats. L-glutamine (1000 mg/kg) treated diabetic rats showed minimal pathological changes may be due to reduction of oxidative stress. L-glutamine offered protection to the heart. Over expression of adhesion molecules may facilitate a leukocyte-endothelial interaction and thus aggravate the inflammatory response and tissue injury while L-glutamine decreases expression of adhesion molecules [9].

In conclusion, administration of L-glutamine effectively controlled the hyperglycemia in stretpozotocin-nicotinamide induced diabetic rats. Supplementation of L-glutamine showed protective effect with respect to electrocardiographic abnormalities, hemodynamic changes and left ventricular contractile function, biological markers of cardiotoxicity, endogenous antioxidant markers. Further histology of heart tissue confirmed the protective role of L-glutamine at cellular level. Role of GLP-1 receptor in cardioprotection is well known [27]. GLP-1 receptor agonist represents a novel approach for the treatment of patients with cardiovascular disease associated with type 2 diabetes [28]. We have reported oral L-Glutamine alone [16] and concomitantly administration with sitagliptin [29] increased active GLP-1 (7-36) amide secretion and improves glycemic control in stretpozotocin-nicotinamide induced diabetic rats. The results obtained in the present investigation demonstrate possible usefulness of L-glutamine is a cardioprotective agent in in streptozotocin-nicotinamide induced diabetic rats by modulation of endogenous antioxidant defense system.
